# The effect of local application of low-magnitude high-frequency vibration on the bone healing of rabbit calvarial defects—a pilot study

**DOI:** 10.1186/s13018-016-0494-7

**Published:** 2016-12-08

**Authors:** Ivan Puhar, Li Ma, Dina Suleimenova, Vasileios Chronopoulos, Nikos Mattheos

**Affiliations:** 1 Department of Periodontology, School of Dental Medicine, University of Zagreb, Zagreb, Croatia; 2 Implant Dentistry, Faculty of Dentistry, The University of Hong Kong, Hong Kong, China

**Keywords:** Animal experiments, Vibration, Biomaterials, Bone substitutes, Guided bone regeneration, Morphometric analysis, Wound healing

## Abstract

**Background:**

The objective of this pilot study was to evaluate the effect of local application of low-magnitude high-frequency vibration (LMHFV) on the bone healing of rabbit calvarial defects that were augmented with different grafting materials and membranes.

**Methods:**

Four calvarial defects were created in each of two New Zealand rabbits and filled with the following materials: biphasic calcium phosphate (BCP), deproteinized bovine bone mineral covered with a non-cross-linked collagen membrane (BO/BG), biphasic calcium phosphate covered with a strontium hydroxyapatite-containing collagen membrane (BCP/SR), and non-cross-linked collagen membrane (BG). Four defects in one rabbit served as a control, while the other was additionally subjected to the local LMHFV protocol of 40 Hz, 16 min per day. The rabbits were sacrificed 1 week after surgery. Histomorphometric analysis was performed to determine the percentages of different tissue compartments.

**Results:**

Compared to the control defects, the higher percentage of osteoid tissue was found in LMHFV BG defects (35.3 vs. 19.3%), followed by BCP/SR (17.3 vs. 2.0%) and BO/BG (9.3 vs. 1.0%). The fraction occupied by the residual grafting material varied from 40.3% in BO/BG to 22.3% in BCP/SR LMHFV defects. Two-way models revealed that material type was only significant for the osteoid (*P*= 0.045) and grafting material (*P* = 0.001) percentages, while the vibration did not provide any statistical significance for all histomorphometric outcomes (*P* > 0.05).

**Conclusion:**

Local application of LMHFV did not appear to offer additional benefit in the initial healing phase of rabbit calvarial defects. Histomorphometric measurements after 1 week of healing demonstrated more pronounced signs of early bone formation in both rabbits that were related with material type and independent of LMHFV.

## Background

Dentoalveolar surgery today is among the most common outpatient surgical treatments routinely conducted in general dentist practice. Although healing after a straightforward extraction is usually uneventful, larger procedures, such as surgical removal of wisdom teeth, can cause significant postoperative pain, swelling, and discomfort for the patient [1, 2].

Bone augmentation techniques include sinus floor elevation, lateral ridge augmentation, distraction osteogenesis, and alveolar ridge preservation [3]. A variety of grafting materials has been used for this purpose, such as autogenous block grafts, allogenic block grafts, xenografts, and alloplastic materials [4]. Guided bone regeneration involves a series of procedures in order to regenerate bone defects, often in conjunction with or in expectation of dental implant placement [5]. After the surgical trauma, slow and complex healing process is initiated in the bone tissue, which involves a sequence of blood clot organization, wound cleansing, tissue formation, and finally, tissue modeling and remodeling [6, 7].

The influence of mechanical stimuli on biological tissue structure and metabolism is crucial aspect of tissue mechanobiology in both healthy and healing tissues [8]. The impact of low-magnitude high-frequency vibration (LMHFV), in particular, has been the subject of many experiments which have pointed out effects on multiple levels, starting from the molecular level, to the cell cultures, and extending to the experiments in animals and humans. Consistently across studies, the application of vibrations showed anabolic and/or anti-catabolic effects in bone [9–12]. Lau et al. confirmed that osteocytes are actually mechanosensors able to detect LMHFV stimulus at the transcriptional level and produce soluble factors that inhibit osteoclast formation [13].

Despite the increasing evidence regarding the whole body vibration, no animal studies have been conducted assessing the effects of locally applied vibration stimuli following a guided bone regeneration surgery. Therefore, the aim of this pilot study was to evaluate the effect of local application of LMHFV on the bone healing of rabbit calvarial defects that were treated with different grafting materials and membranes.

## Methods

Two New Zealand rabbits (age 6 months, weight 3.5–4.0 kg) were used in the study. The rabbits were checked for their health and taken care of by a veterinarian at the Laboratory Animal Unit of the Faculty of Medicine, the University of Hong Kong. The study protocol was approved by the Committee on the Use of Live Animals for Teaching and Research, the University of Hong Kong (CULATR 3058-13). All care and surgical procedures were delivered according to the standards set by the latest Guidelines by the Committee (CULATR), the University of Hong Kong.

The surgical procedure described by Yip et al. was adopted [14]. The rabbits received preoperative analgesics subcutaneously (buprenorphine 0.05 mg/kg). General anesthesia was achieved using ketamine (35 mg/kg), acepromazine (1 mg/kg), and xylazine (5 mg/kg) administered intramuscularly. The scalp covering frontal and parietal bones was shaved and disinfected with 1% iodine solution. After making a midsagittal incision, a full-thickness flap was reflected to expose the calvarium. Under saline irrigation, four critical size defects [15] were marked with a trephine (diameter 7 mm) and finalized with a piezoelectric surgery tip (Implant Centre 2; ACTEON Group, MERIGNAC Cedex, France). Special care was taken to prevent damage of the dura mater.

The eight defects (four in each group) were augmented as follows:

1. Defect 1 (left side of the frontal bone) was filled biphasic calcium phosphate, a mixture of >90% β-tricalcium phosphate and <10% hydroxyapatite with a particle size of 250–1000 μm (Revisios B.V., Bilthoven, the Netherlands) (BCP). The defect was not covered with a barrier membrane.
  BCP is alloplast or synthetically derived graft material.
2. Defect 2 (right side of the frontal bone) was filled with deproteinized bovine bone mineral with a particle size of 250–1000 μm, covered with a non-cross-linked collagen membrane (BioOss/BioGide, Geistlich Pharma AG, Wolhusen, Switzerland) (BO/BG).
  BioOss is produced from the mineral part of bovine bone, while BioGide is a non-cross-linked porcine-derived collagen membrane.
3. Defect 3 (left side of the parietal bone) was filled with biphasic calcium phosphate (Revisios B.V., Bilthoven, the Netherlands) covered with a strontium hydroxyapatite-containing collagen membrane (BCP/SR).
  The heat cross-linked strontium hydroxyapatite-containing collagen membranes were prepared according to the procedures described by Hao et al. [16].
4. Defect 4 (right side of the parietal bone) was covered with non-cross-linked collagen membrane (BioGide, Geistlich Pharma AG, Wolhusen, Switzerland) (BG), while no filler was used.
  Finally, the scalp was sutured with interrupted sutures.

One rabbit served as a control, while the other was subjected to local application of LMHFV of 40 Hz, 3 g, for 16 min per day, for 7 days by direct contact of the vibration device to the calvarial area neighboring to the wounds. The application of the LMHFV took place under anesthesia.

Subcutaneous antibiotics (enrofloxacin 5–10 mg/kg for 3 days) and analgesics (buprenorphine 0.05 mg/kg for 3 days, and thereafter, meloxicam 0.2 mg/kg every 24 h) were administered postoperatively. A veterinarian monitored the health condition and recovery until the time of sacrifice. The rabbits were euthanized 1 week after surgery by an overdose of pentobarbital (150 mg/kg).

The calvarium that contained all four craniotomies was harvested in blocks from both rabbits. The samples were fixed with 10% paraformaldehyde solution and decalcified in 12.5% ethylenediaminetetraacetic acid. After dehydration and embedding in paraffin, sections of 4 μm were made through the middle of the defects and then stained with hematoxylin–eosin.

**Fig. 1:**
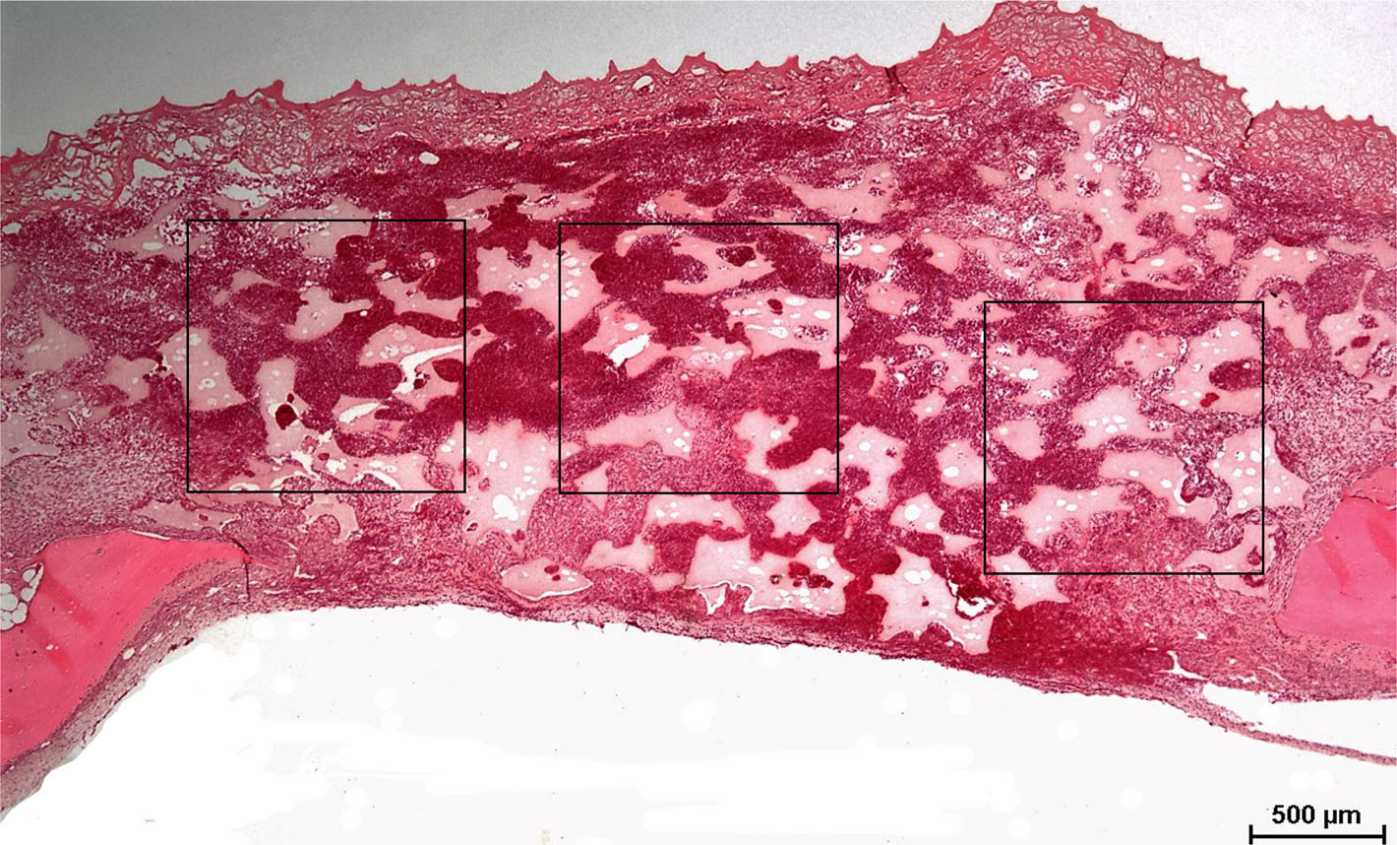
Left, central, and right area were used for histomorphometric analysis (original magnification ×50)

**Fig. 2:**
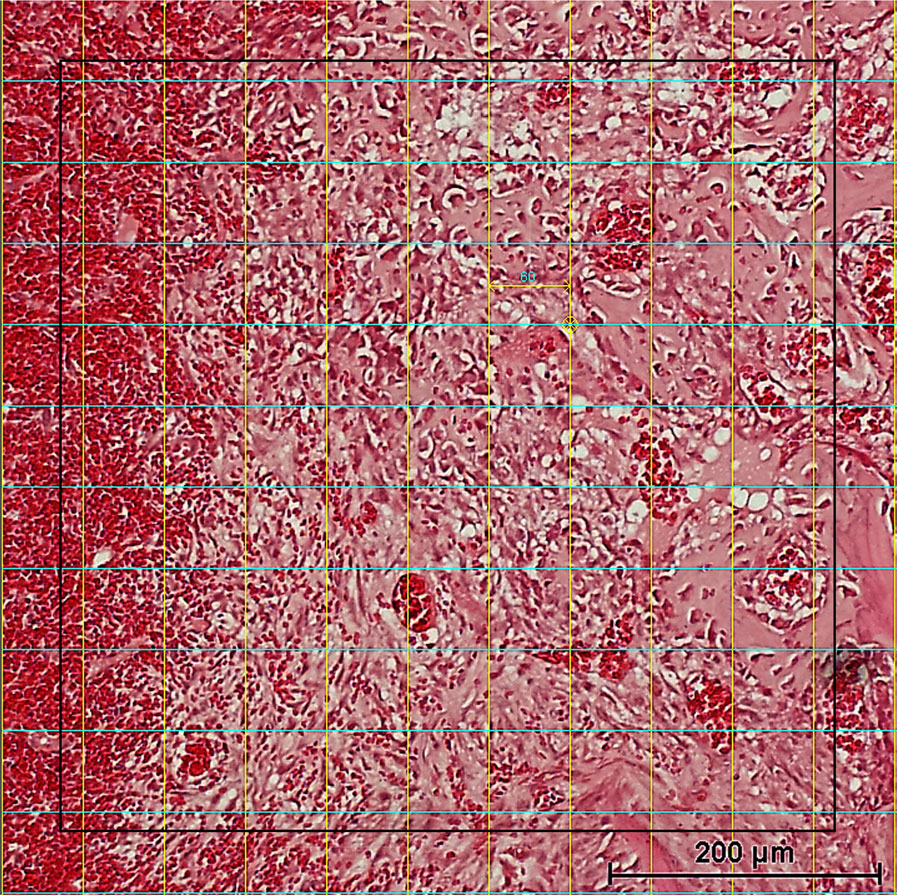
Identification of different tissue types using 10 × 10 grid points

The defects were examined using a light microscope (Nikon® Eclipse VL100POL, Tokyo, Japan) incorporated into a digital video camera (Nikon® Digital Sight DS-Ri1). Images were analyzed with NISElements AR 3.00 software (Nikon® Laboratory Imaging software, Japan). Each defect was observed in three areas: left, central, and right (Fig. 1). Histomorphometric analysis was performed under higher magnification adopting the methods described by Schroeder and Münzel-Pedrazzoli [17]. A square with 10 × 10 grid points (60 μm distance) was placed over the tissues in the middle of each observed area (Fig. 2). Four tissue types were identified as follows:

- Osteoid (OS)
- Fibrous tissue (FB)
- Blood clot (BL)
- Residual grafting material (GR)

The volumetric density (%) of different tissue structures was calculated as an average percentage obtained from all three defect areas. In order to investigate the effects of vibration and material type on each histomorphometric outcome (osteoid, fibrous, blood, and graft), two-way models were performed with a Bonferroni-adjusted post-hoc analysis. Since the sample was limited, no interaction effect could be calculated and only main effect models were performed.

## Results

Both rabbits showed an uneventful healing without any signs of postoperative complications during the 1 week of follow-up.

**Fig. 3:**
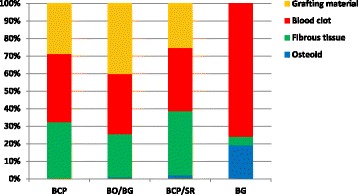
Comparison of different tissue percentages for the control defects. BCP = biphasic calcium phosphate; BO**/**BG = deproteinized bovine bone mineral covered with a non-cross-linked collagen membrane; BCP**/**SR = biphasic calcium phosphate covered with a strontium hydroxyapatite-containing collagen membrane; BG = non-cross-linked collagen membrane

**Fig. 4:**
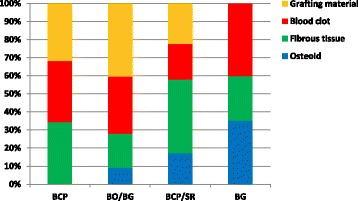
Comparison of different tissue percentages for the experimental defects. BCP = biphasic calcium phosphate; BO**/**BG = deproteinized bovine bone mineral covered with a non-cross-linked collagen membrane; BCP/SR = biphasic calcium phosphate covered with a strontium hydroxyapatite-containing collagen membrane; BG = non-cross-linked collagen membrane

Table 1 and Figs. 3 and 4 show the average percentages of various tissues in the control and experimental rabbit among different materials. Examination under the light microscope revealed osteoid tissue originating from the borders of the BO/BG, BCP/SR, and BG defects. Compared to the control defects, a higher percentage of osteoid tissue was found in experimental defects of BG (35.3 vs. 19.3%), followed by BCP/SR (17.3 vs. 2.0%) and BO/BG (9.3 vs. 1.0%). No osteoid was found in the control and experimental BCP defects. Osteoid was never observed in the central part of the defect (percentages not presented here), meaning no signs of an early bone bridging were noticed.

**Table 1:** Percentages of various tissues in control and experimental defects among different materials

	BCP (%)		BO/BG (%)		BCP/SR (%)		BG (%)	
	Control	Vibration	Control	Vibration	Control	Vibration	Control	Vibration
Osteoid (OS)	0.0	0.0	1.0	9.3	2.0	17.3	19.3	35.3
Fibrous tissue (FB)	32.3	34.3	24.3	18.7	36.7	40.7	4.7	24.7
Blood clot (BL)	39.0	34.0	34.3	31.7	36.0	19.7	76.0	40.0
Grafting material (GR)	28.7	31.7	40.3	40.3	25.3	22.3	0.0	0.0

The fraction occupied by the residual grafting material varied from 40.3% in BO/BG to 22.3% in BCP/SR experimental defects. Higher percentage of the fibrous tissue was found in all experimental defects compared to the controls, except for BO/BG (18.7 vs. 24.3%). Compared to other experimental sites, the highest percentage of blood clot could be identified in BG (40%).

Two-way models revealed that material type was only significant for the osteoid (*P* = 0.045) (observed power = 0.660) and graft material (*P* = 0.001) (observed power = 1.000) percentage, while the vibration did not provide any statistical significance for all histomorphometric outcomes (*P* > 0.05) (Tables 2 and 3). Bonferroni-adjusted pairwise comparison could not show which pairs of material comparisons were significant for osteoid formation (*P* < 0.05). Also, BG had significant lower grafting material values than BCP (*P* = 0.002), BO/BG (*P* = 0.001), and BCP/SR (*P* = 0.005) while BCP/SR had significant lower graft values than BO/BG (*P* = 0.015).

**Table 2:** Relationship between osteoid values and the independent variables in the full model

Independent variables	Estimate	S.E.	*P* value	Observed power
Material type			0.045	0.660
BCP (1)	–27.30	5.28	0.014	
BO/BG (2)	–22.15	5.28	0.025	
BCP/SR (3)	–17.65	5.28	0.044	
BG (4)	0			
Vibration			0.077	0.446
No	–9.90	3.73	0.077	
Yes	0			
*Intercept*	32.25	4.17	0.004

**Table 3:** Relationship between grafting material values and the independent variables in the full model

Independent variables	Estimate	S.E.	*P* value	Observed power
Material type			0.001	1.000
BCP (1)	30.20	1.73	<0.001	
BO/BG (2)	40.30	1.73	<0.001	
BCP/SR (3)	23.80	1.73	0.001	
BG (4)	0			
Vibration			1.000	0.050
No	–7.105E-15	1.23		
Yes	0			
*Intercept*	1.776E-14	1.37	1.000	

## Discussion

During this pilot study, one experimental rabbit was subjected to local application of LMHFV for 1 week after the grafting surgery, while the other was used as a control. The main findings showed a more advanced maturation of both non-grafted defects, with evidence of osteoid tissue in the margins of the defects. This observation was not however matched in the other grafted defects, although the amount of osteoid percentage appeared consistently higher in the vibrated defects. Despite these observations, it has to be noted that LMHFV was not shown as a statistically significant variable for any of the histomorphometric outcomes.

Such findings are therefore not consistent with the increasing evidence of the different biological mechanisms that might modulate the vibration-induced healing response. Osteocytes, osteoblasts, and mesenchymal progenitor cells are the most important mechanosensitive cells that recognize and respond to forces [18]. In a study on human mesenchymal stromal cells, Kim et al. found that daily exposure to vibrations increased the proliferation of human mesenchymal stromal cells into osteoblasts, with the highest efficiency occurring at a peak of 30 to 40 Hz [19]. The frequency of 40 Hz, which was previously proven highly effective, was used in our study as well.

In contrast to other studies, we used LMHFV locally on the calvarial area, while the usual protocol in the earlier experiments was to subject the animal to whole body LMHFV [20, 21].

Different healing outcomes were observed among four biomaterials or their combinations in this study (*P* < 0.05), but a post-hoc analysis could not reveal which pairs of material comparisons are more significant. Previous studies have also reported that different biomaterials produce different results in the early phase of healing when used for bone regeneration [22, 23]. This fact has been shown in a recent study where the gene expression of early healing was studied with different biomaterials [24]. Analysis of both control and experimental defects showed the best results when only a membrane was used with no filler. This is consistent with our understanding that biomaterials used for bone augmentation delay the healing and maturation of bone tissue [25]. Similar healing pattern can be found in some animal studies with various biomaterials and duration of follow-up, although they did not observe the effect of LMHFV [26]. A histomorphometric study in the rabbit cranial vault showed higher mean bone fraction after 1 week in sites treated with deproteinized bovine bone compared to membrane only, control sites. However, in the following weeks, the authors noticed more intense bone growth in the control sites but the differences were not statistically significant. Artzi et al. studied resorption rate and healing morphology of inorganic bovine bone in the canine [27]. After 3 months, they reported significant acceleration of bone formation at the defects covered with membrane only vs. defects with inorganic bovine bone.

The present results also showed that collagen membrane alone holds certain osteoconductive properties. Osteoconductive potential of collagen membrane was histologically proven for the first time in a study by Taguchi et al. [28]. Some of the outcomes regarding the influence on biological mechanisms in osteogenic cells are the following: the membrane allowed the newly formed bone to reach the same height as the pristine bone; immunohistochemistry for alkaline phosphatase, osteopontin, and osteocalcin suggested the induction of osteoblastic differentiation; collagen fibers from the membrane were connected with the new bone matrix adjacent to the membrane [28].

Different tissue percentages found in BCP vs. BCP/SR defects might indicate the importance of the membrane in the early bone healing of grafted sites, given that no osteoid was found in both control and experimental BCP defects. Kitayama et al. showed that strontium hydroxyapatite-containing collagen membrane was as effective as BG for the defect healing if it was supported by the grafting material [29]. In addition, more mineralized new bone can be expected in defects grafted with BCP than in those grafted with BO, which is comparable with our percentages in BCP/SR vs. BO/BG. Similar healing patterns were also demonstrated in a recent pilot study on guided bone regeneration with different biomaterials using rabbit model [24]. Gene expression analysis showed that the strontium hydroxyapatite-containing collagen membrane, combined with either BO or BCP, leads to accelerated bone formation during the early healing phase.

There were no available studies for our comparison that aimed to assess the effect of LMHFV following bone grafting surgery. However, many authors attempted to simulate certain dentoalveolar procedures in animal models and examined the influence of simultaneously applied mechanical stimuli. Morphometric analysis of bone healing after extraction of incisor in rats and applying LMHFV revealed significantly greater trabecular thickness compared with control rats [30]. Furthermore, several authors have explored the influences of vibration on peri-implant bone healing and implant integration in osteoporotic rat models [31–34]. Encouraging results with this impaired animal model repeatedly demonstrated accelerated implant osseointegration, enhanced bone volume around titanium implants, and even a partial reversal of the negative effects of osteoporosis. Higher percentages of osteoid tissue observed in our study, when defects were subjected to LMHFV protocol, can only be explained with material type and not with LMHFV. It is also possible that the significance for LMHFV was not reached due to inadequate sample size (lower power of the study).

The findings of this animal pilot study should be interpreted with caution. The major limitation was a small sample size, with only two rabbits being observed for 1 week. The artificially created calvarial defects were contained, thus favoring bone regeneration, while everyday clinical scenarios usually include more challenging non-contained or combined bone defects. Nevertheless, the current experiment investigated locally applied LMHFV, which may also be considered as a convenient way of application in humans. Given the encouraging evidence from the tissue and animal studies and the experience with the rabbit calvaria model, the authors believe that future experiments could further produce evidence with regards to the role of LMHFV in bone wound healing.

## Conclusions

Based on our result, we cannot conclude that local application of LMHFV provides additional benefit in the initial healing phase of rabbit calvarial defects. After 1 week of healing, histomorphometric measurements demonstrated more pronounced signs of early bone formation in both rabbits that were related with material type and independent of LMHFV. Further studies with larger sample size and different follow-ups are required to better understand the impact of LMHFV on healing of the bone defects treated with various grafting materials and membranes.

## Abbreviations

BCP: Biphasic calcium phosphate
BCP/SR: Biphasic calcium phosphate covered with a strontium hydroxyapatite-containing collagen membrane
BG: Non-cross-linked collagen membrane
BL: Blood clot
BO/BG: Deproteinized bovine bone mineral covered with a non-cross-linked collagen membrane
FB: Fibrous tissue
GR: Residual grafting material
LMHFV: Low-magnitude high-frequency vibration
OS: Osteoid
